# Semicircular canal drug delivery safely targets the inner ear perilymphatic space

**DOI:** 10.1172/jci.insight.173052

**Published:** 2024-11-08

**Authors:** Jinkyung Kim, Jesus Maldonado, Dorothy W. Pan, Patricia M. Quiñones, Samantha Zenteno, John S. Oghalai, Anthony J. Ricci

**Affiliations:** 1 Department of Otolaryngology, Stanford University School of Medicine, Stanford, California, USA.; 2 Caruso Department of Otolaryngology – Head and Neck Surgery, University of Southern California, Los Angeles, California, USA.; 3 Department of Molecular and Cellular Physiology, Stanford University School of Medicine, Stanford, California, USA.

**Keywords:** Otology, Therapeutics, Drug therapy, Gene therapy, Transport

## Abstract

Effective, reproducible, and safe delivery of therapeutics into the inner ear is required for the prevention and treatment of hearing loss. A commonly used delivery method is via the posterior semicircular canal (PSCC); however, its specific targeting within the cochlea remains unclear, impacting precision and reproducibility. To assess safety and target specificity, we conducted in vivo recordings of the pharmacological manipulations delivered through the PSCC. Measurements of auditory brainstem response (ABR), vibrometry, and vestibular behavioral and sensory-evoked potential (VsEP) revealed preserved hearing and vestibular functions after artificial perilymph injections. Injection of curare, a mechanoelectrical transducer (MET) channel blocker that affects hearing when in the endolymph, had no effect on ABR or VsEP thresholds. Conversely, injection of CNQX, an AMPA receptor blocker, or lidocaine, a Na^+^ channel blocker, which affects hearing when in the perilymph, significantly increased both thresholds, indicating that PSCC injections selectively target the perilymphatic space. In vivo tracking of gold nanoparticles confirmed their exclusive distribution in the perilymph during PSCC injection, supporting the pharmacological finding. Together, PSCC injection is a safe method for inner ear delivery, specifically targeting the perilymphatic space. Our findings will allow for precise delivery of therapeutics within the inner ear for therapeutic and research purposes.

## Abstract 2

Semicircular canal injection is a safe method for delivering drugs or genes to the inner ear and specifically targets the perilymphatic space

## Graphical abstract

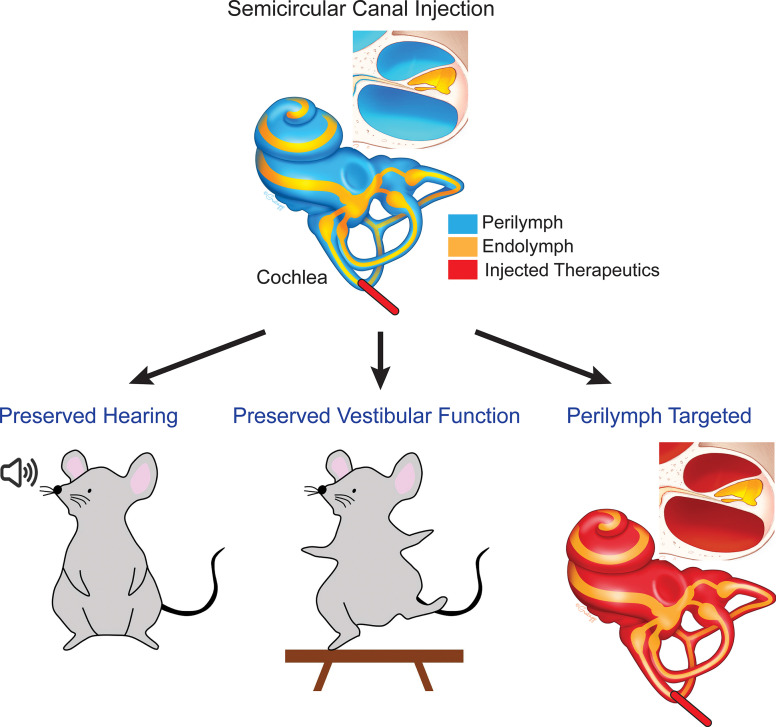

## Introduction

Hearing loss is one of the most common disabilities caused by mechanical trauma, loud sounds, aging, infection, chemotoxicity, and genetic defects. Approximately 15% of adults in the United States suffer from some degree of hearing loss (1). The number of hearing loss patients is on the rise and is expected to be 73.5 million (22.6%) by 2060 (2). A leading cause of hearing loss is cochlear hair cell degeneration (3). This degenerative process is irreversible because mature mammalian cochlear hair cells do not regenerate (4). Thus, there is a need for effective and safe delivery of therapeutics to the inner ear for prevention and treatment of hearing loss.

Selective and precise targeting of the inner ear is crucial for drug delivery, as it enhances therapeutic effects and reduces off-target effects (5). Nonspecific targeting necessitates higher drug dosages than required, which can result in adverse effects due to off-target binding. However, accessing the inner ear is challenging due to several obstacles, including (i) the inner ear is deeply located within the temporal bone and is surrounded by neighboring structures; (ii) it is a mechanically sensitive structure enclosed in bone and filled with fluids, making direct access to the inner ear risky and potentially leading to hearing and/or vestibular dysfunction (6–8); (iii) the blood-labyrinth barrier (BLB) acts as a physical barrier and filters blood-borne compounds; and (iv) it is a small volume consisting of 2 separate compartments, with limited communication between them. Consequently, the development and testing of a drug delivery approach is a critical step in establishing an effective therapeutic pathway to the inner ear.

**Figure 1:**
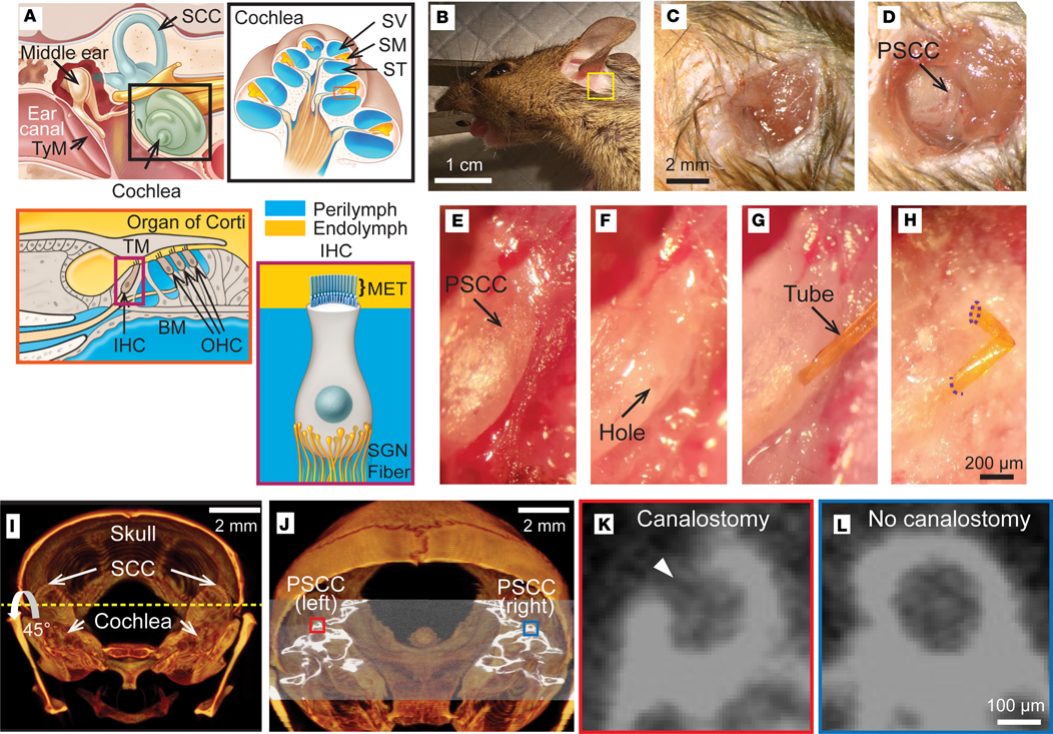
Surgical approach for PSCC injection into the cochlea. The zoomed-in images correspond to the identically colored boxes. (**A**) Illustrations of external, middle, and inner ear (top left), cochlea (top right), organ of Corti (bottom left), and inner hair cell (bottom right). TyM, tympanic membrane; SCC, semicircular canal; SV, scala vestibuli; SM, scala media; ST, scala tympani; MET, mechanoelectrical transducer channel; SGN, spiral ganglion neuron; IHC, inner hair cell; OHC, outer hair cell; TM, tectorial membrane; BM, basilar membrane. (**B**) Head-fixed mouse is laterally rotated to access the left posterior semicircular canal (PSCC). (**C**) Skin below the pinna is excised to reach the PSCC. (**D**) The backward L-shaped tissue orienting the position for dissection to the PSCC. (**E**) The PSCC is exposed after removing subcutaneous fat and muscle. (**F**) A hole is formed in the PSCC after PAG treatment. (**G**) A microtube is snuggly inserted through the hole for PSCC injection. (**H**) After the injection, the microtube is trimmed, folded, and attached to neighboring tissue with cyanoacrylate glue. Purple dotted lines indicate the location where the glue is applied. (**I**) 3D volume rendered image of microcomputed tomography shows the skull, including cochleae and SCCs. (**J**) The cross-sectional image of the PSCC with (red box) and without (blue box) the canalostomy with 45° rotation around the axis (yellow dotted line in panel **I**). The magnified cross-sectional images of the PSCC showing (**K**) a hole from the canalostomy (arrowhead) and (**L**) an intact canal from the non-surgery condition. Scale bars: 1 cm (**B**), 2 mm (**C**), 200 μm (**E**–**H**), and 100 μm (**K** and **L**).

Sound waves passing through the ear canal cause vibrations in the tympanic membrane. These vibrations are then transmitted to the middle ear ossicles and ultimately result in the movement of the stapes footplate, which connects to the oval window of the cochlea (9) (Figure 1A). Vibration of the oval window creates a pressure wave between the scala media (SM) and scala tympani (ST) that leads to motion of the basilar membrane (BM) (Figure 1A). The sensory hair cells located on the BM are stimulated by the vibrations of this membrane in relation to the tectorial membrane (TM). This stimulation leads to the shearing of the sensory hair bundle, resulting in the opening and closing of mechanoelectrical transducer (MET) channels (Figure 1A). The apical surface of the sensory hair cells, including the sensory hair bundles with the MET channels, is located in the endolymph-filled SM compartment. The remaining portion of the hair cell, which forms synapses with the spiral ganglion neurons, resides in the perilymphatic space, comprising 2 compartments, the ST and scala vestibuli (SV) (Figure 1A). Depending on their location, cochlear cells are exposed to different fluid compartments: (i) the endolymphatic space, such as cells in the stria vascularis; (ii) the perilymphatic space, like spiral ganglion neurons; or (iii) both, such as sensory hair cells that have their apical surface bathed in endolymph, while their basolateral surface is bathed in perilymph. When targeting drugs, it is crucial to consider which cells and even which specific parts of the cell are most accessible for effective delivery.

The endolymphatic space is a closed compartment containing a high potassium (K^+^) and low calcium (Ca^2+^) solution (10, 11). The ionic milieu of the endolymph is maintained by transport through the stria vascularis and Reissner’s membrane, which may also transport pharmacological agents into this space (12, 13). Drug targeting through the blood supply would have to traverse the stria vascularis and/or Reissner’s membrane to enter the SM. In contrast, perilymph composition is like cerebrospinal fluid. Depending on age and species, perilymph exits the cochlea at the cochlear aqueduct and thus has access to the brain. Perilymph is replenished via the BLB (5). This barrier is selective and so drug delivery to the perilymph via blood will strongly depend on its chemical structure as to how well it traverses the BLB.

A variety of delivery strategies have been tested to target therapeutics to the inner ear. Systemic delivery through the bloodstream is easy and noninvasive; however, it can induce off-target effects and potentially be rapidly cleared by the kidney, liver, or immune system (5, 14). The BLB is a major hurdle, as it can restrict the entry of therapeutic compounds into the inner ear, resulting in lower drug levels at the target site (5, 15, 16). Blood delivery also means that the compounds might be transported through the stria vascularis or across Reissner’s membrane, so each new compound needs to be tested for access to inner ear fluids and to identify the final compartment reached. External and middle ear deliveries such as directly into the ear canal or transtympanically are limited in that the therapeutics can be cleared through the Eustachian tube. The round window membrane (RWM) may also be a diffusion barrier to delivery. A recent study demonstrated that intratympanic injection followed by cochlear pumping promotes an even distribution of drugs along the entire cochlea (17). An advantage to these local approaches is there should be fewer systemic off-target effects (18, 19). Intracochlear delivery is more targeted to the inner ear, resulting in a higher drug concentration within the inner ear while minimizing systemic distribution of the drug. The RWM and posterior semicircular canal (PSCC) have been used as injection routes to directly deliver therapeutics to intracochlear spaces. RWM injection needs to be accessed through the middle ear after opening the tympanic bulla, often leading to hearing loss (20–23). The injection is often not uniformly distributed and can create a basal-to-apical gradient (24–27). Furthermore, the injected markers are often detected in the brain, submeningeal, or spinal cord regions (25, 28, 29). RWM injection may be shunted via the cochlear aqueduct depending on the delivery rate (25). PSCC injection does not require opening the tympanic bulla, minimizing surgical damage and resulting in better preservation of hearing function (23, 30, 31). Also, experimental and modeling data demonstrate that the injection results in a uniform distribution within the cochlea (25, 32).

Here, we examined PSCC injection as a strategy for inner ear drug delivery. Our work specifically addresses 3 critical questions: (i) whether PSCC injection causes damage to either auditory or vestibular function, (ii) whether the injection targets the endolymphatic and/or perilymphatic space, and (iii) whether the delivery is consistent and reproducible. Our in vivo investigations of auditory brainstem responses (ABR), vestibular sensory-evoked potential (VsEP), vestibular function assessment using behavioral tests, and vibrometry using optical coherence tomography (OCT), combined with the pharmacological dissection, revealed that the PSCC injection strategy is a safe method to deliver therapeutics into the inner ear. The injections reproducibly and effectively target the perilymphatic space.

## Results

### A canalostomy for the PSCC injection into the cochlea.

We performed a canalostomy on the PSCC to allow for fluid injections into the cochlea. Figure 1A provides a schematic of the middle and inner ear for orientation of the components of interest. The hemicochlea shows the multiple compartments and the unique positioning of the sensory hair cells and some supporting cells between fluid compartments. Also, the schematic shows the location of the semicircular canal (SCC), including the PSCC, as a site for inner ear delivery without the need for invasive opening. An adult mouse (4–8 weeks old) was head fixed (33, 34) and laterally rotated to the left side to access the PSCC (Figure 1B). Skin below the pinna was incised to reach the PSCC bone (yellow box in Figure 1, B and C). The PSCC was exposed after removing subcutaneous fat and muscle with a cauterizer (Figure 1, D and E). A hole was created on the PSCC (diameter ≈ 110 μm) (Figure 1F). Before creating the hole, a phosphoric acid gel (PAG) was applied to thin the bone (33, 34). This step minimized the mechanical stress while creating the hole, allowing the position and size of the hole to be precisely controlled. The chemo-mechanical strategy also prevented accidental bone breaking. A polyimide microtube was snuggly inserted into the PSCC for the intracochlear injection (Figure 1G) (35). After the injection, the microtube remained inserted to avoid backflow and was trimmed to a short length (~1 mm) (Figure 1H). The resealing step folded the tube stump and attached it to the neighboring tissue with cyanoacrylate glue (dotted lines in Figure 1H). The glue was also used to close the incised skin. To better visualize the position and size of the canalostomy, we performed microcomputed tomography scans. Generating 3D-rendered volumes identified the skull and both cochleae, including the SCCs (Figure 1I). The cross-sectional image with 45° rotation shows the PSCC with (red box) and without (blue box) the canalostomy (Figure 1J). A hole for the microtube insertion was successfully created on the PSCC (arrowhead in Figure 1K) when compared with the nonsurgical side (Figure 1L). We evaluated the success of the surgery by checking for any fluid leakage after the resealing step (Figure 1H). If leakage was observed, often caused by a larger hole (diameter > 120 μm), an additional crack on the PSCC, or improper resealing, we excluded the mouse from the study (~11%). In later experiments where we used a micromanipulator for tube insertion, we prevented damage to the PSCC and reduced the time from canalostomy to tube insertion down to seconds (Supplemental Figures 1 and 2; details in Methods).

### Hearing function is preserved after artificial perilymph injection through the PSCC.

**Figure 2:**
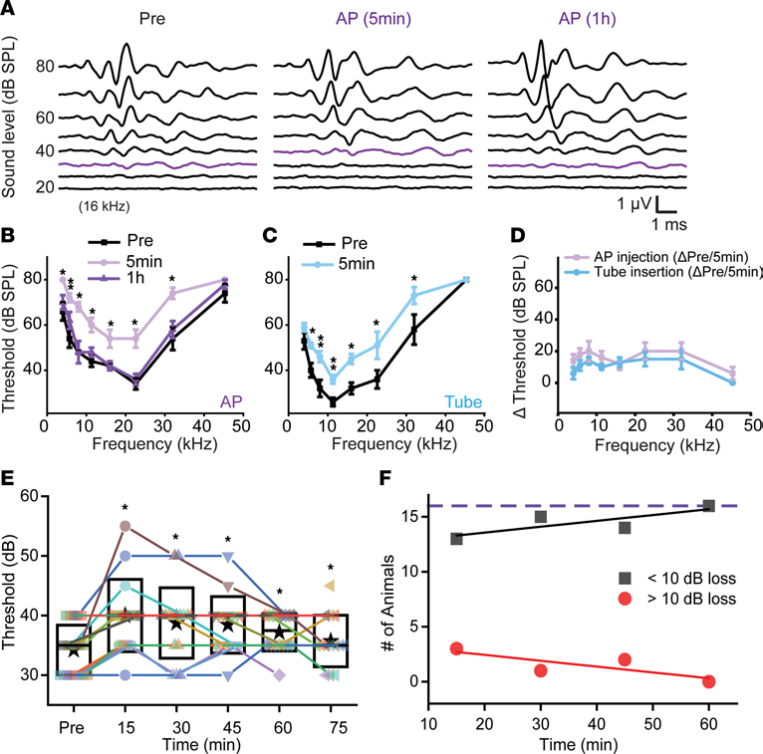
Auditory brainstem response (ABR) after artificial perilymph injection through the PSCC. (**A**) Representative ABR responses at 16 kHz before, 5 minutes after, and 1 hour after artificial perilymph (AP) injection. Purple lines indicate thresholds of the ABR responses. (**B**) ABR thresholds before, 5 minutes after, and 1 hour after AP injection (*n* = 5). While the thresholds increased 5 minutes after the injection as compared with preinjection (*P < 0.05, **P < 0.01 by paired, 2-tailed Student’s *t* test), no statistically significant shifts were observed between preinjection and 1 hour after the injection (*P* > 0.05 by paired, 2-tailed Student’s *t* test). (**C**) ABR thresholds before and 5 minutes after tube insertion (i.e., no injection) (*n* = 5). *P < 0.05, **P < 0.01 by paired, 2-tailed Student’s *t* test. (**D**) Threshold changes before and 5 minutes after injection (panel **B**: light purple line) and tube insertion (panel **C**: light blue line). *P* > 0.05 by unpaired, 2-tailed Student’s *t* test. (**E**) Individual animal responses to click ABRs measured before and every 15 minutes after injection. Boxes, SD; stars, mean; lines, median (*n* = 15). *P < 0.002, 0.003, 0.001, 0.004, and 0.03 for the 15, 30, 45, 60, and 75 minute time points, respectively, by paired, 2-tailed Student’s *t* test. (**F**) Plots the number of animals showing greater than 10 dB (red) or less than 10 dB (black) threshold shifts after injection against time after injection. By 60 minutes, no animals had greater than 10-dB shifts.

PSCC injection has been reported to result in no to mild hearing loss (23, 30, 36–39). Here, we examined hearing function closely at time points from 5 minutes to 2 weeks after artificial perilymph injection (1 μL, injection speed 100 nL/sec) by measuring the ABR. Representative ABR waveforms at 16 kHz reveal that the threshold 5 minutes after injection was slightly elevated, but returned to the preinjection level within 1 hour (Figure 2A). The average threshold in the 5-minute-postinjection group was higher than that in the preinjected group (0.001 < *P* < 0.05, paired *t* test) (Figure 2B). However, it recovered to the preinjection level within 1 hour (*P* > 0.05, paired *t* test) (Figure 2B). The immediate temporary threshold shift (TTS) was induced by the microtube insertion into the PSCC, shown by ABR threshold elevation after the microtube insertion without injection (Figure 2C) (0.001 < *P* < 0.05, paired *t* test). The threshold changes in the 5-minute-postinjected group compared to the preinjected group were not statistically different compared to the microtube-inserted group (*P* > 0.05, unpaired *t* test) (Figure 2D). We further investigated the time course of recovery after modifying the injection system to allow for faster placement of the injecting tube by using a micromanipulator. We found that there were still some slight threshold shifts, but all recovered within 60 minutes of injecting (Figure 2, E and F). Figure 2E shows individual animals’ responses to click stimulation. We probed these data with 5-dB increments in sound pressure levels (SPLs), revealing a small statistical difference after injection using a paired *t* test. However, only 3 of 15 animals had threshold shifts greater than 10 dB SPL and in each case these animals recovered by 60 minutes (Figure 2F). Most animals showed no or a less than 10-dB shift, ostensibly due to the faster placement reducing leakage of cochlear fluid (39).

**Figure 3:**
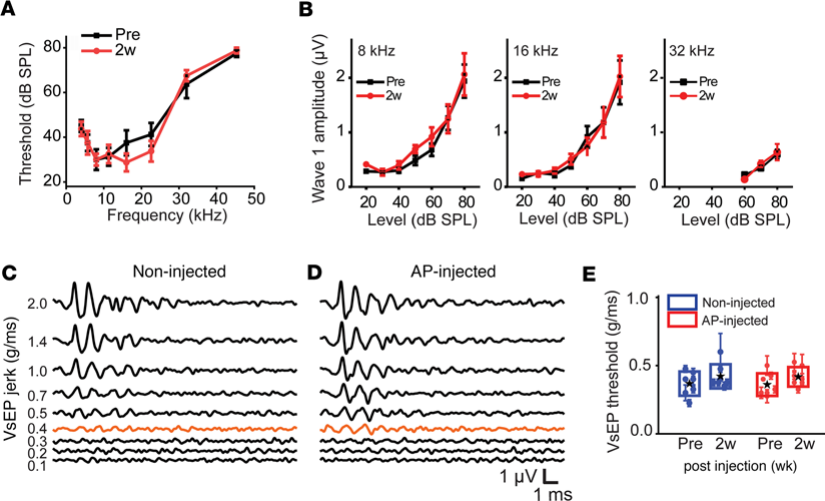
Auditory brainstem responses (ABR) and vestibular-evoked potentials (VsEP) after artificial perilymph injection through the PSCC reveal no long term effects. (**A**) ABR thresholds in preinjection and 2-week-postinjection conditions (*n* = 8). *P* > 0.05 by paired, 2-tailed Student’s *t* test. (**B**) ABR wave 1 amplitude at 8, 16, and 32 kHz in preinjection and 2-week-postinjection conditions (*n* = 8). *P* > 0.05 by paired, 2-tailed Student’s *t* test. (**C**) Representative VsEP responses in non-injected mice (left ear; non-surgery) and (**D**) 2 weeks after artificial perilymph (AP) injected mice (right ear). Orange lines indicate thresholds of the VsEP responses. (**E**) VsEP thresholds in non- (*n* = 7; 4 females and 3 males) and AP-injected (*n* = 7; 4 females and 3 males) groups with preinjection and 2-week-postinjection conditions. The thresholds are not significantly different between preinjection and 2-week-postinjection conditions (*P* > 0.05 by paired, 2-tailed Student’s *t* test) as well as non- and 2-week-postinjection conditions (*P* > 0.05 by unpaired, 2-tailed Student’s *t* test). No statistical difference between sexes was also detected (*P* > 0.05 by unpaired, 2-tailed Student’s *t* test). The error bars indicate the variation of the threshold decisions by 4 researchers. Boxes are SD; stars are the means across animals.

We next assessed long-term hearing function after the PSCC injection. Two weeks after injection of artificial perilymph, the ABR threshold was unchanged from control measurements (*P* > 0.05, paired *t* test) (Figure 3A). We also measured the peak-to-peak amplitude of ABR wave 1 for all stimulus intensities at 8, 16, and 32 kHz as a proxy for cochlear synaptopathy (40). There were no differences between the preinjected and 2-week-postinjected groups (*P* > 0.05, paired *t* test) (Figure 3B). Therefore, the PSCC injection to the cochlea induced no hearing defect. Our data suggest that the PSCC injection is a safe method to deliver therapeutics to the inner ear.

### Vestibular function is preserved after artificial perilymph injection through the PSCC.

Given that the injection is via the PSCC and the microtube is left embedded and sealed within the SCC, it was critical to assess vestibular function. We tested function before and 2 weeks after injection of artificial perilymph by assessing behavioral functions associated with balance disorders. We also used VsEP, which represents compound action potentials elicited by linear acceleration in the naso-occipital axis (41–43). Age- and sex-matched noninjected mice were tested as a control for the injected mice. Two weeks after artificial perilymph injection through the PSCC (1 μL, injection speed 100 nL/sec), no behavioral defect was identified as measured using head bobbing, head tossing, head tilting, circling, trunk-curl, or swim test (Supplemental Videos 1 and 2, and Supplemental Figure 3). Note that the scores of the swimming tests were zero (i.e., normal swimming) for both groups based on criteria previously reported (44). Consistent with the behavioral test results, the injected mice uniformly displayed robust VsEP thresholds similar to the noninjected mice (Figure 3, C and D). The average response in the injected group (0.418 ± 0.027 g/ms) was similar to that prior to injection (before, 0.361 ± 0.031 g/ms) and the noninjected group (2 weeks, 0.421 ± 0.033 g/ms) (Figure 3E) (*P* > 0.05, paired *t* test between preinjection and 2-week-postinjection conditions and unpaired *t* test between non- and injected groups). Thus, our data identify no vestibular defect induced by the PSCC injection. The caveat with these data is that the VsEP is biased toward the otolith organs and less toward the SCC. Future work will need to assess the vestibular ocular reflex to directly test SCC function.

### Cochlear mechanics were preserved after artificial perilymph injection through the PSCC.

**Figure 4:**
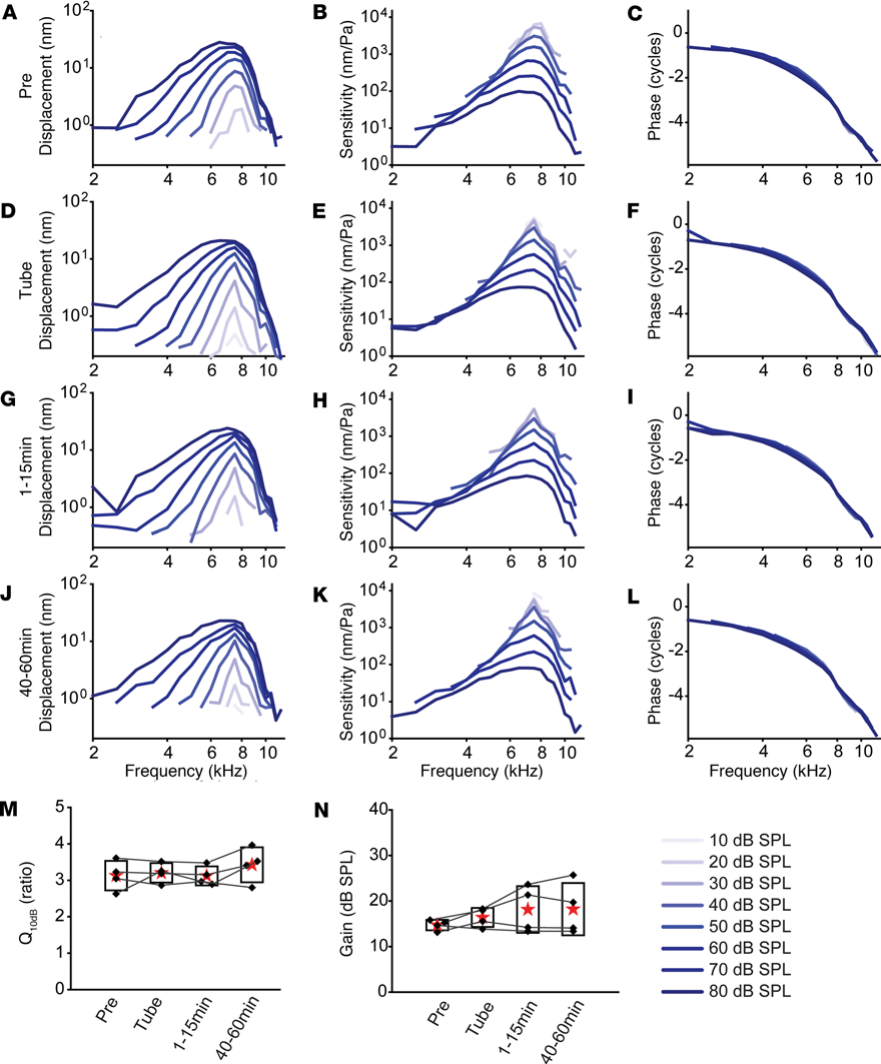
Cochlear mechanics after artificial perilymph injection through the PSCC. (**A**–**L**) Tuning curves, sensitivity curves, and phase measurements recorded at the tectorial membrane using 10- to 80-dB SPL sound stimuli with 2–15 kHz pure tones. Representative curves (**A**–**C**) at baseline, (**D**–**F**) after canalostomy, (**G**–**I**) 1–15 minutes after artificial perilymph injections, and (**J**–**L**) 40–60 minutes after artificial perilymph injection. (**M**) Q_10dB_ measurements calculated using the 40-dB tuning curves (*n* = 4). *P* > 0.05 by paired, 2-tailed Student’s *t* test. (**N**) Cochlear gain calculated between the 30- and 80-dB SPL tuning curves. No significant differences exist between all conditions (*n* = 4). *P* > 0.05 by paired, 2-tailed Student’s *t* test.

We further tested how PSCC injection affected cochlear mechanics using OCT to measure sound-induced vibrations from the tectorial membrane. We measured both the magnitude and phase of the motion in response to a range of stimulus frequencies and intensities, and analyzed the data as previously described (45–47). As an example, we present data from 1 representative mouse. Prior to opening the inner ear, sound-stimulated vibrations demonstrated a normal tuned response, with higher intensity stimuli producing larger vibrations (Figure 4A). The response magnitudes peaked at the best frequency of the cochlear recording location. The best frequency decreased slightly as the stimulus intensity increased, as expected. We then calculated the sensitivity by dividing the vibratory magnitude by the stimulus intensity (Figure 4B). Sensitivity is used to characterize the large degree of nonlinear gain in the motion response, as is expected in a healthy mouse cochlea. Lower intensity stimuli were amplified more than higher intensity stimuli. The phase of the response is measured relative to the stimulus phase and its slope, known as the group delay, shows the delay in traveling wave propagation along the length of the cochlea. The shape of the phase responses demonstrated a frequency-dependent lag consistent with normal traveling wave propagation (Figure 4C). Next, we opened the PSCC and inserted the injection tube. There were no gross changes in the vibratory magnitudes, sensitivity, or phase responses stemming from this manipulation (Figure 4, D–F). Similarly, repeated measures initially after injecting artificial perilymph (Figure 4, G–I) or 40–60 minutes later (Figure 4, J–L) demonstrated no obvious changes in vibrational patterns. To assess the impact of our procedures on the most sensitive feature of cochlear function, cochlear amplification, we measured vibrometry at baseline, after tube insertion, and 1–15 minutes and 40–60 minutes after artificial perilymph injection in a cohort of 4 mice. The Q_10dB_, a measure of tuning sharpness, was calculated by determining the frequency of the largest vibratory magnitude using the 40-dB SPL stimulus level and dividing by the width of the amplitude curve, also known as the bandwidth 10 dB below this peak (Figure 4M). The average Q_10dB_ was 3.13 ± 0.41 at baseline, 3.20 ± 0.27 after canalostomy and tube insertion, 3.12 ± 0.26 at 1–15 minutes after artificial perilymph injection, and 3.42 ± 0.48 at 40–60 minutes after artificial perilymph injection. There were no differences between these measurements (*P* > 0.05, paired *t* test). These data show that the sharpness of tuning, or Q_10dB_, is unchanged by canalostomy and artificial perilymph injection. We also quantified gain, a direct measurement of the amount of amplification. We calculated gain from the tectorial membrane displacement between the 30- and 80-dB SPL stimulus levels at the best frequency (Figure 4N). The gain was 14.7 ± 1.2 dB SPL at baseline, 16.4 ± 2.1 dB SPL after canalostomy and tube insertion, 18.1 ± 5.1 dB SPL at 1–15 minutes after artificial perilymph injection, and 18.2 ± 5.7 dB SPL at 40–60 minutes after artificial perilymph injection. There were no differences in gain at baseline, after canalostomy and tube insertion, or injection of artificial perilymph (*P* > 0.05, paired *t* test). These data further confirm that cochlear mechanics are not negatively affected by PSCC injection and that it is a safe method for cargo delivery to the cochlea.

The OCT data in Figure 4, D–I might appear to contradict the ABR data in Figure 2 where a TTS was observed. However, the explanation is simply a technical issue, where animal positioning for OCT measurements following injection took longer to set up so that the vibrometry early measure did not happen until 15 minutes after the injection, a time point where the thresholds had returned to near normal. Supplemental Figure 4 provides a diagram of the timeline for the experiments to better make this point.

### Pharmacological manipulations revealed that PSCC injection entered the perilymphatic, not the endolymphatic, space.

**Figure 5:**
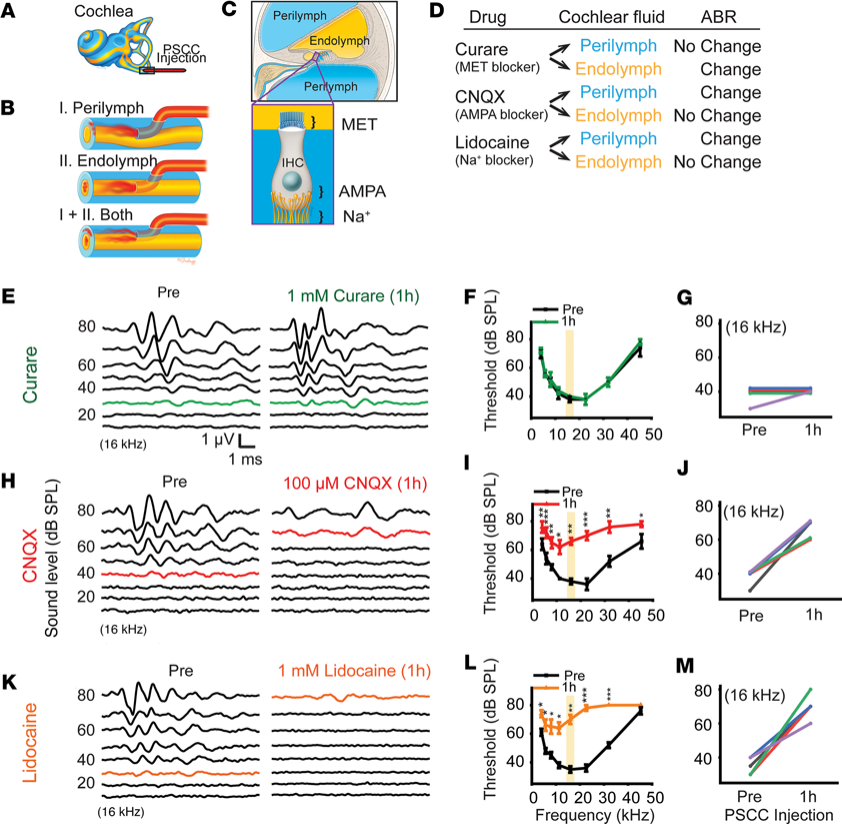
Auditory brainstem response (ABR) after pharmacological manipulations through the PSCC. (**A**) Illustration of cochlea with the PSCC injection, where sky blue represents perilymph and yellow represents endolymph. (**B**) The zoomed-in images of the box in panel **A** showing possible entry pathways of the PSCC injection, to the perilymph (top), endolymph (middle), or both perilymph and endolymph (bottom). (**C**) The cross-sectional image of the cochlea shows perilymph and endolymph-filled chambers. The magnified image of the box shows the locations of mechanoelectrical transducer (MET) channel in the inner hair cell (IHC), AMPA receptor at the level of the IHC basolateral membrane, and the Na^+^ channel in the nerve fibers that synapse with the IHC. (**D**) The table shows the predicted drug effect on ABR results depending on the compartment (perilymph or endolymph) of entry. (**E**) Representative ABR responses at 16 kHz in preinjection and 1 hour after curare injection. Green lines indicate thresholds of the ABR responses. (**F**) ABR thresholds in preinjection and 1 hour after curare injection (*n* = 5). *P* > 0.05 by paired, 2-tailed Student’s *t* test. (**G**) ABR thresholds of each individual mouse at 16 kHz in preinjection and 1 hour after curare injection (yellow box in panel **F**). (**H**) Representative ABR responses at 16 kHz in preinjection and 1 hour after CNQX injection. Red lines indicate thresholds of the ABR responses. (**I**) ABR thresholds in preinjection and 1 hour after CNQX injection (*n* = 5). *P < 0.05, **P < 0.01, ***P < 0.001 by paired, 2-tailed Student’s *t* test. (**J**) ABR thresholds of each individual mouse at 16 kHz in preinjection and 1 hour after CNQX injection (yellow box in panel **I**). (**K**) Representative ABR responses at 16 kHz in preinjection and 1 hour after lidocaine injection. Orange lines indicate thresholds of the ABR responses. (**L**) ABR thresholds in preinjection and 1 hour after lidocaine injection (*n* = 5). *P < 0.05, **P < 0.01, ***P < 0.001 by paired, 2-tailed Student’s *t* test. (**M**) ABR thresholds of each individual mouse at 16 kHz in preinjection and 1 hour after lidocaine injection (yellow box in panel **L**).

Having to this point demonstrated that the canal injection delivery pathway was safe for both vestibular and auditory function, we next identified which cochlear compartment(s) were injected and whether the delivery location was consistent. Targeting and reproducibility are critical for establishing a rigorous and reproducible delivery system. For example, therapeutics need to enter the SM (endolymph) to target stereocilia, reticular lamina, or stria vascularis, while they need to enter the ST (perilymph) to target the BM, hair cell soma, synapses, and spiral ganglion neuron fibers (48–50). However, it remains unclear which cochlear compartment PSCC injection targets. PSCC injection can target (i) perilymphatic, (ii) endolymphatic, or (iii) both spaces, and if both it might be variable (Figure 5, A and B). Three drugs were injected through the PSCC (1 μL, injection speed 100 nL/sec) to identify the site of delivery (Figure 5, C and D). The drug effect was determined by ABR recordings prior to and 1 hour after injections. Curare (tubocurarine), which is a MET channel blocker, was tested first (51, 52). MET channels are located at the tips of hair cell stereocilia that project into the SM (endolymph) (53) (Figure 5C), so curare will block MET channels, resulting in elevated ABR thresholds if it enters the SM (Figure 5D). The second drug, 6-cyano-7-nitroquinoxaline-2,3-dione (CNQX), is an AMPA receptor blocker, and the last drug lidocaine is a sodium (Na^+^) channel blocker. Both AMPA receptors and Na^+^ channels are located postsynaptically on spiral ganglion cell processes that reside in the ST (perilymph) (Figure 5C); if the ABR threshold is elevated with CNQX and lidocaine, then it indicates PSCC injection into perilymphatic space (Figure 5D). The difference between CNQX and lidocaine would be local diffusion barriers associated with a postsynaptic location, as compared with a location along the distal dendrite.

One hour after 1 mM curare injection, representative ABR waveforms at 16 kHz revealed the same threshold in pre- and postinjected groups (Figure 5E). The average threshold in the 1-hour-postinjected group was similar to that in the preinjected group (*P* > 0.05, paired *t* test) (Figure 5F). Curare injection did not elevate ABR thresholds despite using a concentration several orders of magnitude higher than its half blocking dose, suggesting that curare did not have access to MET channels located in the endolymph compartment (51). Four of 5 animals revealed no threshold shift after curare injection, suggesting that the delivery was reliably not to endolymph (Figure 5G). In contrast, both 100 μM CNQX and 1 mM lidocaine injections induced large threshold shifts, as demonstrated in the representative ABR waveforms at 16 kHz (Figure 5, H and K). The average thresholds in 1-hour-postinjected groups were significantly elevated as compared with preinjected groups (*P* < 0.01, paired *t* test) (Figure 5, I and L). The elevation of ABR thresholds by CNQX and lidocaine for every mouse tested suggests that the drugs delivered via PSCC injections were consistently reaching the perilymphatic space (Figure 5, J and M). We conclude that PSCC injection exclusively enters the perilymphatic space.

### In vivo OCT imaging of nanoparticles confirmed PSCC injection targets the perilymphatic space.

**Figure 6:**
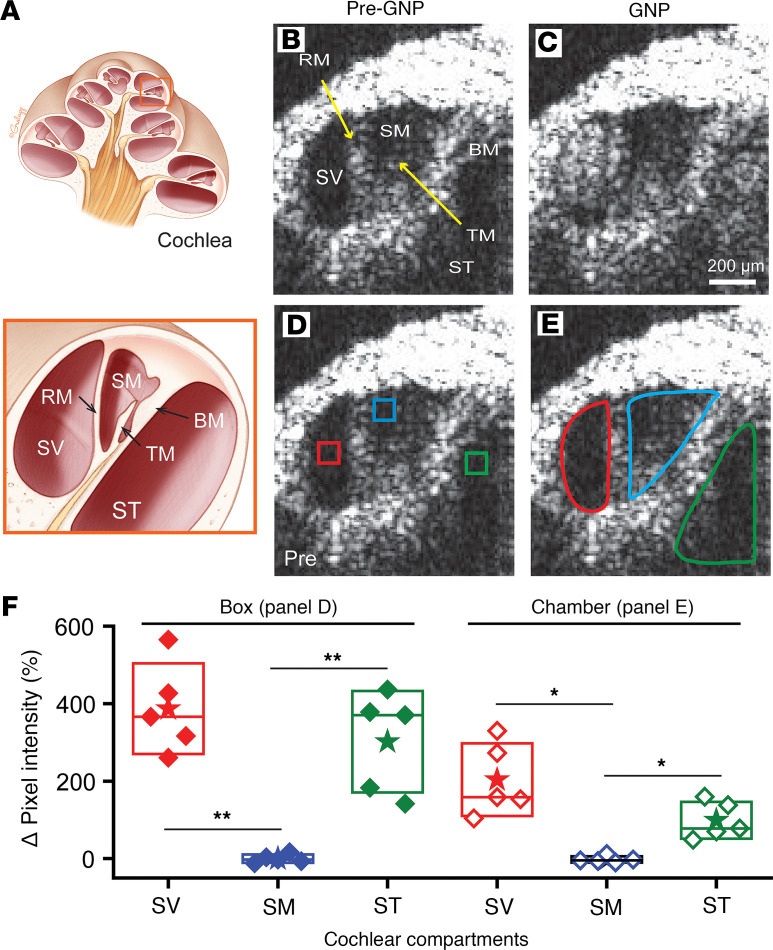
In vivo real-time monitoring of gold nanoparticles (GNPs) injected through the PSCC. (**A**) The top presents the cross-sectional image of the cochlea and the bottom shows the region outlined in orange rotated to match the OCT images. Cochlear chambers are labeled: scala vestibuli (SV), scala media (SM), and scala tympani (ST). These chambers are separated by Reissner’s membrane (RM) and basilar membrane (BM). Tectorial membrane (TM) is within the SM compartment. Optical coherence tomography (OCT) images with the same labeling as panel **A** show (**B**) before GNP (50 nm) injection and (**C**) after GNP injection. (**D**) Boxes show small regions of interest (ROIs) that avoid the organ of Corti tissue structure. (**E**) Entire chamber labeling includes the organ of Corti structures. Scale bar: 200 μm (**B**–**E**). (**F**) The change in fluorescence intensity with GNP injection. Closed symbols are box ROIs from panel **D** and open symbols are the entire chamber ROIs from panel **E**. Boxes, SD; stars, mean; lines, median. *P < 0.05; **P < 0.01 by paired, 2-tailed Student’s *t* test. Two-way ANOVA with Tukey’s post hoc test also revealed significant differences between the SM and SV, as well as between the SM and ST, in both the box and chamber regions.

To further investigate the targeting of the PSCC injection, we imaged gold nanoparticles (GNPs, 50 nm) being injected through the PSCC in live mice using OCT (54). In vivo OCT imaging resolved 3 cochlear chambers, the SV, SM, and ST (Figure 6, A and B). We performed time-lapse imaging to localize the GNPs while injecting (Supplemental Video 3) and compared the images obtained before the injection with those captured during the injection (Figure 6, B and C). The injected GNPs filled both the SV and ST (perilymphatic space), whereas the SM (endolymphatic space) remained clear (Figure 6C). To quantify this, we measured the pixel intensity in the boxes (Figure 6D) and the entire chambers (Figure 6E) before and after GNP injections (red, blue, and green in the SV, SM, and ST, respectively). We selected small regions of interest (boxes) to exclude pixel intensities from the organ of Corti tissues (Figure 6D), and also analyzed regions encompassing the entire chambers (Figure 6E). Summary data in Figure 6F show in both cases that the pixel intensity within the SV and ST increased; however, no significant change was observed within the SM (boxes, *P* < 0.01; chambers, *P* < 0.05; paired *t* test). We never observed a mixed response; nanoparticles only appeared in perilymphatic space. Thus, in vivo real-time imaging supports the pharmacological assessment that PSCC injection exclusively enters the perilymphatic space in a very reproducible way.

**Figure 7:**
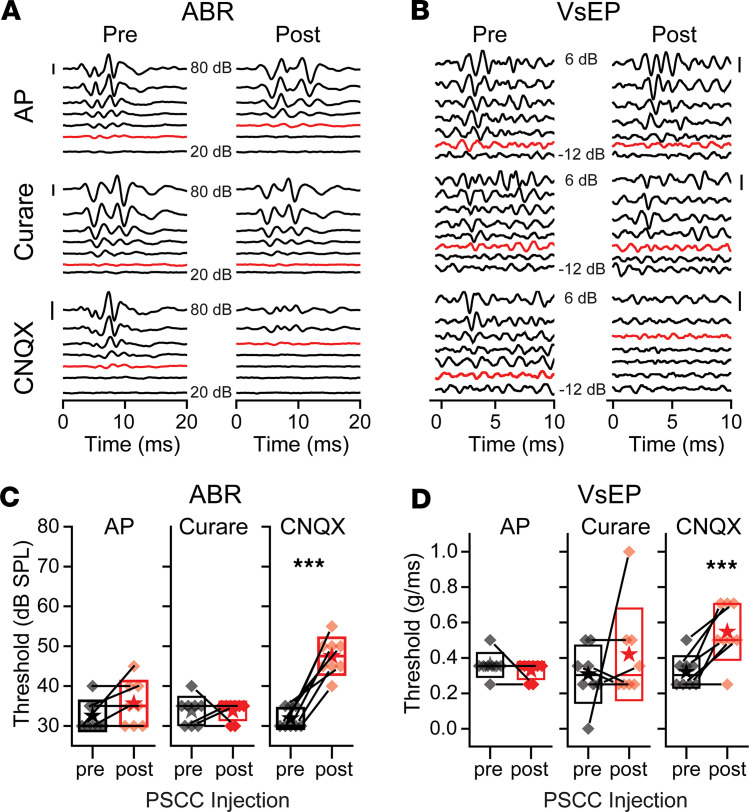
Auditory brainstem responses (ABR) and vestibular-evoked potentials (VsEP) after artificial perilymph, curare, or CNQX injections through the PSCC. (**A**) Representative traces of ABR recordings 1 hour after injection of artificial perilymph (AP), curare (1 mM), or CNQX (100 μM). Responses are shown for stimulus intensity from 20 to 80 dB SPL in 10-dB SPL increments. (**B**) Representative traces of VsEP measurements from the same animals as in panel **A** with stimuli varying between 0.1 and 2.0 g/ms. Red traces indicate thresholds. Scale bars: 1 μV. Summary data for (**C**) ABR (*n* = 8) and (**D**) VsEP (*n* = 8) show preinjection (black) and 1 hour after injection (red) symbols for individual animals. Boxes, SD; stars, mean; lines, median. ***P < 0.001 by paired, 2-tailed Student’s *t* test for the CNQX treatment for both ABR and VsEP.

As a final test for characterizing PSCC drug delivery, we measured both ABR and VsEP in mice after delivery of curare or CNQX. It is plausible that curare may enter endolymph for the vestibular system, but not the cochlea. Potentially, the narrowness of ductus reunions can limit flow into the cochlea, as compared to the vestibular organs. Representative examples of the ABR and the VsEP traces are presented in Figure 7, A and B for artificial perilymph (control), 1 mM curare, or 100 μM CNQX injection. One hour after the injection, only the CNQX group elevated thresholds for both ABR and VsEP measurements. Summary data also present that only CNQX evoked a statistical difference for both ABR and VsEP (Figure 7, C and D). With CNQX injection, all animals showed an increase in threshold for ABR and all but 1 animal showed an increase in VsEP threshold. Both artificial perilymph and curare injections showed a consistent trend in ABR and VsEP thresholds before and after drug delivery. Together, these data support the argument that drug delivery targets the perilymph compartment.

## Discussion

**Figure 8:**
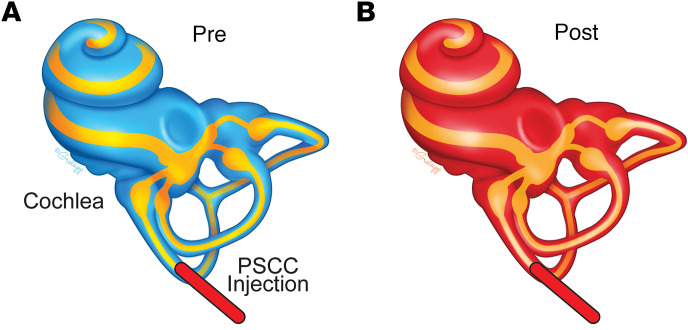
PSCC injection targets the perilymphatic space. (**A**) Illustration demonstrates the cochlea with perilymph (sky blue) and endolymph (yellow). (**B**) Illustration reveals the PSCC injection (red) enters the perilymphatic, not endolymphatic, space.

Our hearing and vestibular function assessments revealed that the PSCC injection strategy is a safe method to deliver therapeutics into the inner ear. Furthermore, in vivo OCT imaging, combined with the pharmacological dissection, found that the injection reproducibly and exclusively targets the perilymphatic space in the inner ear (Figure 8).

Therapeutic delivery into the inner ear is essential for the prevention and treatment of hearing loss. Here, we examined the PSCC injection method, focusing on safety, targeting, and reproducibility. Correct and reproducible targeting of therapeutics is critical to any successful therapy. As the cochlea is partitioned into different physical compartments with separate routes of access and clearance, it is essential that drugs be targeted to the appropriate compartment. Behavioral, electrophysiological, and OCT measurements suggest that the PSCC delivery approach is safe. Pharmacological dissection of the delivery route supports the hypothesis that PSCC injections enter exclusively into the perilymphatic space (Figures 5 and 7). OCT imaging of GNPs entering the perilymphatic space during PSCC injection further confirms the perilymphatic space as the target of PSCC delivery (Figure 6).

Previous studies reported that PSCC injection induces ABR threshold elevations approximately ranging from 0 to 30 dB SPL (39, 55–57). We found that ABR thresholds 2 weeks after the PSCC injections were not significantly changed compared to the preinjection baseline (*P* > 0.05, paired *t* test). One potential reason for the discrepancy may be the surgical approach. Mechanical stress during the canalostomy can affect hearing and vestibular functions. Our chemo-mechanical approach prevents unexpected bone cracking and ensures the hole size is consistent. A smaller hole does not allow for proper insertion of the microtube, while a bigger hole increases leakage of intracochlear fluid, which can lead to loss of function. We applied a PAG gel to thin the bone before creating the hole (33, 34). The additional step minimized the mechanical stress and precisely controlled the size and position of the hole. The resealing process after hole formation is also important to preserve function. The consistency in hole size achieved through our chemo-mechanical approach enabled us to properly reseal the hole, resulting in improved preservation of hearing and vestibular functions.

Our ABR recordings demonstrate that PSCC injection of artificial perilymph did not induce hearing loss 2 weeks after injection (Figure 3, A and B). Both thresholds and wave 1 peak-to-peak amplitudes of the ABR were unaffected 2 weeks after injection, indicating no loss of mechanical sensitivity or synapse numbers. The PSCC injection induced a TTS in the ABR measurement that returned to normal within 1 hour after the injection (Figure 2) and was due to microtube insertion alone (Figure 2, B–D), suggesting a change in the pressure wave induced at insertion could be responsible for the TTS. It is likely that slower insertion or introducing a pressure release valve would alleviate the TTS. Supporting this idea, when we switched to a robotic control of the tube insertion, which reduced the time from hole opening to insertion and reduced the pressure associated with inserting the tube, the TTS was reduced (Figure 2, E and F, and Supplemental Figures 1 and 2). However, there were no meaningful differences in the shape of the tuning curves, nor the Q_10dB_ at 40 dB SPL, nor the cochlear gain between 30 and 80 dB SPL (Figure 4). Perhaps OCT cochlear mechanics measurements that focus on more subtle changes, like outer hair cell operating point, are warranted (47). An alternative explanation is that due to the nature of the techniques, OCT vibrometry measurements after canalostomy and tube insertion have a longer lag time than that for ABR measurements. The longer time required for rotation and repositioning of the mouse for vibrometry measurements could result in cochlear mechanics returning to baseline by the time measurements were made, as compared with ABR measurements that are immediately recorded after canalostomy and injection. Supplemental Figure 4 shows the timeline of both experiments, and this might explain the apparent discrepancy. Taken together, these experiments show that cochlear mechanics do not change with canalostomy and artificial perilymph injection. This is consistent with stable ABRs after artificial perilymph injection at 1 hour. Therefore, the PSCC injection technique is a safe, hearing-preserving technique for delivering therapeutics.

A caveat to the conclusion that PSCC injection is safe is that we tested vestibular function using the VsEP, which targets otolith organs over the semicircular canal. The behavioral tests did not identify abnormal responses, but these results could have been masked by central compensatory mechanisms. It is possible, and future testing will determine whether the vestibular ocular reflex is affected. This is particularly worth testing because we leave the tube inserted, simply seal the end, and close the canal opening. We tried removing the tube, but this led to backflow, which compromised the accuracy of injection volume and led to elevated thresholds. Additionally, our testing was in adult mice, and much work has been done in neonatal mice. With neonatal mice, a glass pipette is inserted through the PSCC cartilage to inject. It remains plausible that the pipette can penetrate the membranous labyrinth, a system of ducts and chambers filled with endolymph, allowing the injected fluid to enter the endolymph. Future work will involve testing the use of a glass pipette in adult animals, with the goal of removing the pipette to prevent vestibular dysfunction and monitoring voltage as a tool to identify the endolymph or perilymph compartment. Lastly, the volume and speed of injection are critical for safe and selective inner ear delivery. High volume and speed can potentially cause the injected fluid to spread to nontargeted regions such as the brain, spinal cord, or the contralateral cochlea (25, 58, 59). We have not tested all potential volumes and rates.

It is rapidly becoming more appreciated that the PSCC approach for drug delivery to mice is superior to round window delivery, providing more uniform delivery to the entire cochlea and having less variability between animals. Will PSCC injection translate to humans? Anatomically, the same obstacles identified in rodents are expected to be present in humans, so the basic solution of using PSCC is likely translatable. However, the surgical approach in humans is more difficult than in mice due to the relative orientation of the inner ear and the thickness of the bone. Drilling is required and there is more risk of damage to other structures like the facial nerve. Significantly better efficacy and reproducibility are necessary for the more difficult approach to be used. In mice, although results are better with PSCC delivery method, the cost benefit in terms of riskier procedure in humans is not yet apparent. Recent clinical trials of inner ear gene therapy in humans used the round window as a delivery route for drug application (60–63). The success of these studies in terms of frequency range recovered will provide data as to whether improved drug delivery systems are needed. Similarly, an argument can be made for testing the chemo-mechanical approach, which greatly reduces variability and damage compared with the mechanical-only surgery. However, human bone is much thicker and may require an unrealistic time for the gel to work. Perhaps a hybrid solution will be found where first the bone is thinned and then the gel is used for the finer dissection. These approaches need to be vetted for larger animals and human application. With the growing need for surgical access to the cochlea due to gene therapy and other pharmacological approaches, the vetting of novel safe access to the inner ear is essential.

The fact that PSCC injection targets only the perilymphatic space does not mean that injected therapeutics remain only in the perilymphatic space long term. A variety of transporters exist between the cochlear compartments that can allow passage of compounds between compartments (64). Thus, transport between the cochlear compartments depends on which pharmacological compound or gene construct is injected. Previous studies show that cochlear genes delivered through the PSCC were detected not only in the perilymphatic space, but also in the endolymphatic space (27, 30), potentially indicating that transport mechanisms across Reissner’s membrane or potentially across the BLB are involved. Either of these mechanisms could be involved and compounds need to be individually tested to determine specificity over time. Moreover, the age of the animal at the time of PSCC injection is crucial in determining the target location of drug delivery, as the BLB is established by postnatal day 14 (5).

These data demonstrate that precise delivery of drugs or genes within the inner ear for therapeutic and research purposes is possible through the PSCC. Future directions include developing a strategy to target the endolymphatic space and developing a slow-release strategy for therapeutics delivered through the PSCC based on an implantable osmotic pump or slow-release gel application. This future work will permit greater targeted therapeutic delivery to the inner ear compartments for hearing protection and treatment.

## Methods

### Sex as a biological variable.

Both male and female mice were used in equal proportions in this study. Therefore, sex was not considered as a biological variable.

### Animals.

We used C57BL/6 (Charles River, 026) and CBA (JAX, 000654) mice of both sexes between 4 and 8 weeks old. No sex differences were observed in any experiments we performed. Mice were anesthetized using ketamine (100 mg/kg) and xylazine (10 mg/kg). After 1 hour, we injected supplemental anesthetic (25% of initial dose) every 30 minutes. Anesthesia level was assessed by signs of movement or withdrawal reflex before the application of supplemental anesthetic.

### Mouse canalostomy.

The procedure was performed under a surgical scope (Leica m220) on a 37°C heating pad while mice were anesthetized. After sterilizing with 70% ethyl alcohol, skin below the pinna was excised (5 mm) to reach the PSCC (Figure 1, B and C). By removing subcutaneous fat and muscle tissue with a cauterizer (Bovie High Temperature Cautery), the PSCC bone was exposed (Figure 1, C–E). To minimize mechanical stress while creating a hole for the injection, we applied PAG (Etching gel, Young) to the PSCC bone for 20 seconds to 1 minute, depending on the moisture level around the bone (34). Moisture reduces PAG activity, requiring longer treatment times in higher moisture environments, so we ensured the area is dry before application. A 1× PBS–soaked Kimwipe was used to remove the remaining PAG. Then, we scraped the softened bone with a needle (30 G × 1/2, BD). After applying PAG and scraping, a hole was created on the PSCC (diameter ≈ 110 μm) using the needle and with minimal mechanical stress (Figure 1F). The soft microtube (Customized, ID 0.0038 in.; OD 0.0048 in.; wall 0.0048 in., MicroLumen) was snuggly inserted into the hole for the PSCC injection (Figure 1G). For the majority of experiments, a micromanipulator (Sutter) was used for positioning the tube into the hole (Supplemental Figure 1). Using the manipulator greatly reduced the time to insertion and limited bending or crimping of the tubing during insertion. After the microtube insertion, the edge of the hole on the bone was sealed with cyanoacrylate glue (Permabond Instant Adhesive 101). After the injection, the microtube remained inserted and was trimmed to a short length (~1 mm). The tube stump was folded and attached to the neighbor tissue with cyanoacrylate glue (Figure 1H). The glue was also used to close the excised skin.

### PSCC injection protocol.

A gas-tight syringe (10 μL, Hamilton) was mounted onto a microinjection pump (UMP3 UltraMicroPump, WPI) coupled to a glass pipette. Two soft microtubes were connected to each other with a glue gun (tube 1: BB31695-PE/B, SCI; tube 2: ID 0.0038 in. OD 0.0048 in., MicroLumen), which were attached to the glass pipette for PSCC injection. The pump was mounted to the micromanipulator with the tubing and delivery system arrangement (Supplemental Figure 1A). Supplemental Figure 1B shows the syringe pipette coupling to the tubing and the micropipette holder adapted to connect tube 1 and then tube 2 used for insertion. Supplemental Figure 1C presents the parts of the tubing holder. The back end squeezes tube 1 around tube 2 to prevent leakage, while the front end uses a glass pipette to hold the insertion tubing. The volume and speed of the injection were determined by a syringe pump (Micro 4, WPI). Pump volume was calibrated by injecting dye into oil (Supplemental Figure 2, A and C). We compared a calibrated pipette volume to that ejected by the micropump; we found no impact of the pump rate (Supplemental Figure 2B). We also found the micropump to be reliable across multiple volumes (Supplemental Figure 2D). The syringe, the glass pipette, and microtubes were filled with degassed mineral oil and then 4 μL of artificial perilymph or drugs was loaded for the PSCC injection. This strategy eliminated compression, allowing for a stable and reproducible injection. One microliter of artificial perilymph was injected (in mM: 142 NaCl, 2 KCl, 2 CaCl_2_, 1 MgCl_2_, 10 HEPES, 6 glucose, 2 ascorbate/pyruvate, 2 creatine monohydrate at pH 7.4 and a final osmolality of 304–307 mOsm). Curare (1 mM) (93750, Sigma-Aldrich), 100 μM CNQX (1045, Tocris), or 1 mM lidocaine (491227, Fresenius Kabi) were made in the same artificial perilymph. The injection speeds were 100 nL/sec (Figures 2, 3, 5, and 7) and 500 nL/min (Figure 4). Note that the highly concentrated 50 nm GNP suspension in 0.1 mM PBS (753645, Sigma-Aldrich) was manually injected by hand, as the micropump could not provide enough pressure to push the highly concentrated GNPs in the narrow microtube. During the procedure, the injection site was closely monitored for leakage. No leakage of fluids was observed during or after the PSCC injection.

### Microcomputed tomography.

Microcomputed tomography (SkyScan 1276, Bruker) was carried out under anesthesia immediately following canalostomy. The scanning protocol involved the Bruker software with the following settings: 4032 × 2688 (resolution), 200 mm (scanning position), and 0.5 ° (rotation step). Exposure time was 612 ms with a pixel size of 10.03 μm. Images were reconstructed into cross-sectional images with the NRecon software (Bruker). Reconstructed slices consist of 4032 × 4032 pixels in the horizontal and vertical directions. The vertically stacked 2-dimensional slices were volume rendered as 3D images using Amira software (Visage Imaging).

### Vestibular function tests.

Vestibular function was assessed with behavioral tests and VsEP responses before and 2 weeks after PSCC injection of artificial perilymph (1 μL, 100 nL/sec). Mice were placed into a plastic container (30 × 19 × 12 cm) and their behavior (head bobbing/tossing/tilting and circling) was observed for 3 minutes with video recordings (44, 65–67). The trunk-curl test was performed by holding the tail and observing whether the mouse reached for a nearby surface or curled toward the base of their tail (44). Lastly, a forced swimming test was evaluated using a plastic container (30 × 19 × 12 cm) filled with water at 24°C–26°C. Swimming ability was evaluated for 1 minute from a video recording and scored 0–3 (44). After the behavioral tests, VsEP responses were recorded under anesthesia (68). Subcutaneous electrodes were placed over the caudal cerebrum (non-inverting), behind the right pinna (inverting), and in the right thigh muscle (ground). A head clip was used to secure the head to the mechanical shaker, which was used to deliver linear vestibular stimuli in the naso-occipital axis. Vertical motion of the shaker was monitored by an accelerometer (1018, Vibra-Metrics) and adjusted to produce the appropriate stimulus waveforms. Jerk stimuli ranging from 0.1 to 2.0 g/ms were applied. We collected the responses of non-inverted and inverted stimulus polarities with 256 sweeps. A free-field speaker (FF1, Tucker-Davis Technologies) created a masker sound (90 dB SPL; bandwidth 50 Hz to 50 kHz) to prevent responses from the auditory components of cranial nerve VIII. Four researchers defined the thresholds by eye as the lowest intensity level at which any peaks were identified.

### ABR measurement.

ABR potentials were recorded with a TDT system (System 3 RZ6, Tucker-Davis Technologies) under anesthesia. Three electrodes were positioned in the vertex of the skull (+), the ventral surface of the tympanic bulla (–), and the hind leg (ground). We applied the stimulus with a 5-ms duration and 0.2-ms gate time with the frequency (4–46.1 kHz) and the intensity (10 to 80 dB SPL; 10-dB SPL steps). Click stimulation was also used to monitor recovery from the surgery (10 to 80 dB SPL; 5-dB SPL steps). Responses were sampled and averaged 512 times at each sound level. The major peaks in the ABR waveforms were tracked and the lowest intensity level at which any of peaks were identified was determined as threshold. For the purpose of averaging, we set the threshold to 80 dB SPL when ABR peaks were not detected. Wave 1 peak-to-peak amplitudes in ABR waveforms were measured at 8, 16, and 32 kHz. Click ABRs were used in Figure 2, E and F and Figure 7 to save time and allow for repeated measures for the former and for VsEP in the latter data sets.

### Calibration of delivery system.

We calibrated the micropump to ensure accurate delivery (Supplemental Figure 2). Trypan blue (0.4% in PBS) was diluted to 1:9 with Milli-Q water. A gas-tight syringe (10 μL, Hamilton) was mounted onto a microinjection pump (UMP3 UltraMicroPump, WPI) coupled to a glass pipette. Two soft microtubes were connected to each other with a glue gun (tube 1, BB31695-PE/B, SCI; tube 2: customized, ID 0.0038 in.; OD 0.0048 in.; wall 0.0048 in., MicroLumen) and were attached to the glass pipette for injection. Trypan blue was injected into light silicone oil, programmed to dispense 1 μL at rates of 0.3, 0.5, and 1.0 μL/min (*n* = 5 at each rate). Then, trypan blue was injected at a rate of 0.5 μL/min with volumes of 0.25, 0.5, 0.75, and 1 μL (*n* = 5 at each volume). For comparison, trypan blue was also pipetted into silicone oil using a calibrated Eppendorf Research Plus pipette (maximum volume, 2.5 μL). Photographs were acquired with a Logitech Brio 4K Webcam attached to the lens of the Olympus dissecting microscope. A photograph of a 2-mm stage micrometer was measured in ImageJ (NIH) to set the scale. The photographs were processed in ImageJ and the radius of each sphere in mm was measured using UCB Vision Sciences’ Hough Circle Transform plugin. Volumes were calculated using *V* = 4/3π*r*^3^, where *r* is the radius. Volumes were corrected by dividing expected volumes by the mode of each pipetted volume and multiplying pipetted and injected volumes by this correction factor.

### OCT and vibrometry.

OCT imaging and vibrometry was performed using a custom-built system that has been previously described (69–71). Briefly, a laser (Insight) of wavelength 1310 nm, bandwidth 90 nm, and sweep rate 100 kHz was mounted on a dissecting stereomicroscope (Carl Zeiss). Sound stimuli for vibrometry measurements were delivered in the open-field configuration by a Fostek speaker and consisted of pure tones ranging from 2 kHz to 15 kHz in 0.5-kHz steps and amplitudes ranging from 10 dB to 80 dB SPL in 10-dB steps. Stimulus duration was 100 msec. At baseline, OCT images of the cochlea were acquired and vibrometry measurements recorded at the tectorial membrane. The mouse was then rotated to visualize the PSCC, and a canalostomy was made in the PSCC with the tip of a needle, as described above. The injection tubing was inserted into the PSCC, and the tubing and canalostomy were sealed with histoacryl glue (Tissue Seal, LLC). The mouse was then rotated to visualize the cochlea, and OCT vibrometry measurements recorded after canalostomy, but prior to artificial perilymph injection. After the injection, serial OCT images alternating with vibrometry measurements were recorded. Tuning curves were plotted as a function of displacement against stimulus frequency. Phase unwrapping was performed for the phase plots. For all figures, we did not plot data where the responses were below the noise floor. The characteristic frequency was identified using the peak vibratory response at the lowest stimulus intensity above the noise floor. Cochlear gain was quantified by comparing the 80 and 30 dB SPL sensitivity at the characteristic frequency, calculated as 20log_10_(displacement at 80 dB SPL/displacement at 30 dB SPL). Tuning curve sharpness was quantified by calculating the Q_10dB_ using the tuning curves at 40 dB stimulus intensity, which was the lowest stimulus intensity cleanly able to calculate Q_10dB_ detectable above the noise floor for all conditions tested. The Q_10dB_ was calculated by dividing the amplitude at the characteristic frequency by the bandwidth 10 dB below the vibratory magnitude at the characteristic frequency.

### Statistics.

Statistical analyses were performed using Excel (Microsoft) and Origin (OriginLab). *P* values of less than 0.05 from 2-tailed Student’s *t* tests were considered statistically significant. Unless otherwise noted, stated error bars represent the standard error of the mean (SEM). *P < 0.05; **P < 0.01; ***P < 0.001; no mark, not significant (*P* > 0.05).

### Study approval.

All studies were carried out according to the protocols approved by the Institutional Animal Care and Use Committee at Stanford University (APLAC-14345) and at the University of Southern California (20724).

## Author contributions

JK and AJR designed the experiments. JK and JM performed the PSCC injection, ABR and VSEP, vestibular behavioral tests, mouse surgery and pharmacological manipulation. SZ and AJR redesigned the drug delivery system and calibrated the pump. JK, DWP, PMQ, and JSO performed mouse surgery, PSCC injection, in vivo OCT imaging, vibrometry, and data analysis. JK, DWP, PMQ, JSO, and AJR wrote the manuscript. AJR supervised the entire project.

## Supplementary Material

Supplemental data

Supplemental video 1

Supplemental video 2

Supplemental video 3

Supporting data values

## Version 1 11/08/2024

Electronic publication
