# Supplementary material for: Semicircular canal drug delivery safely targets the inner ear perilymphatic space
Source: JCI Insight. 2024 Nov 8;9(21):e173052. doi: 10.1172/jci.insight.173052 (PMC11601569; doi:10.1172/jci.insight.173052)
Supplement: Supplemental data [file jciinsight-9-173052-s315.pdf]

Supplemental data

Semicircular Canal Drug Delivery Safely Targets Inner Ear Perilymphatic Space

Jinkyung Kim[1,$], Jesus Maldonado[1], Dorothy W. Pan[2], Patricia M Quiñones[2], Samantha Zenteno[1], John S. Oghalai[2], Anthony J. Ricci[1,3,*]

[1]Department of Otolaryngology, Stanford University School of Medicine, Stanford, CA, USA
[2]Caruso Department of Otolaryngology – Head and Neck Surgery, University of Southern California, Los Angeles, CA, USA
[3]Department of Molecular and Cellular Physiology, Stanford University School of Medicine, Stanford, CA, USA

*Correspondence: Anthony J. Ricci
Maile address: 240 Pasteur Drive, Biomedical Innovation Building 0500, Stanford CA 94304
Phone number: 650-736-1290
Email address: aricci@stanford.edu

[$] present address: Department of Otolaryngology, Washington University School of Medicine, St. Louis, MO, USA

Supplemental Figure 1

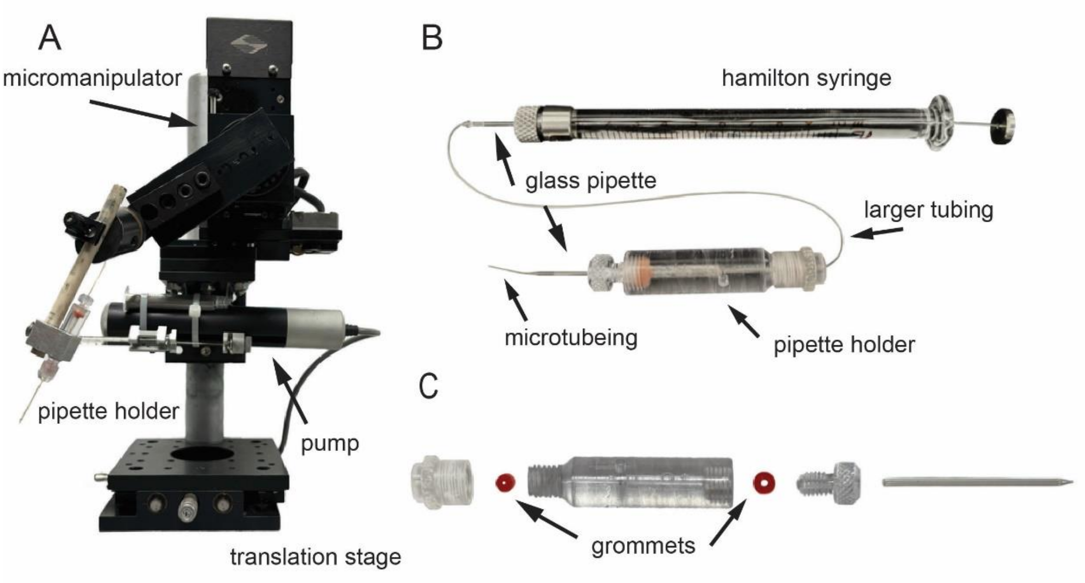

Sfigure1. Apparatus for delivering controlled volumes to the PSCC. (**A**) A Sutter micromanipulator equipped with a pipette holder that is used to support the tube that is inserted into the PSCC. The pump is attached to the manipulator stand so that it moves with the manipulator. The entire apparatus is mounted on an XY translation stage (Newport) to provide additional extent of motion to the system. (**B**) Magnified view of the Hamilton syringe and tubing connection to a standard patch electrode holder. The smaller tubing (SCI, cat. no. BB31695-PE/B) is threaded through the pipette holder back to the syringe. The back end of the pipette holder is tightened to seal the 2 tubings together. The front end of the pipette holder has a glass pipette into which the smaller tubing is threaded. The glass provides stability to the thinner tubing (MicroLumen cat. no. 039-I) while it is inserted. (**C**) Separated components of the pipette holder showing the grommet (orange) at the back end that squeezes the tubings together and the grommet at the front end that seals the glass pipette into the holder, allowing all fluid to flow through the tubing.

Supplemental Figure 2

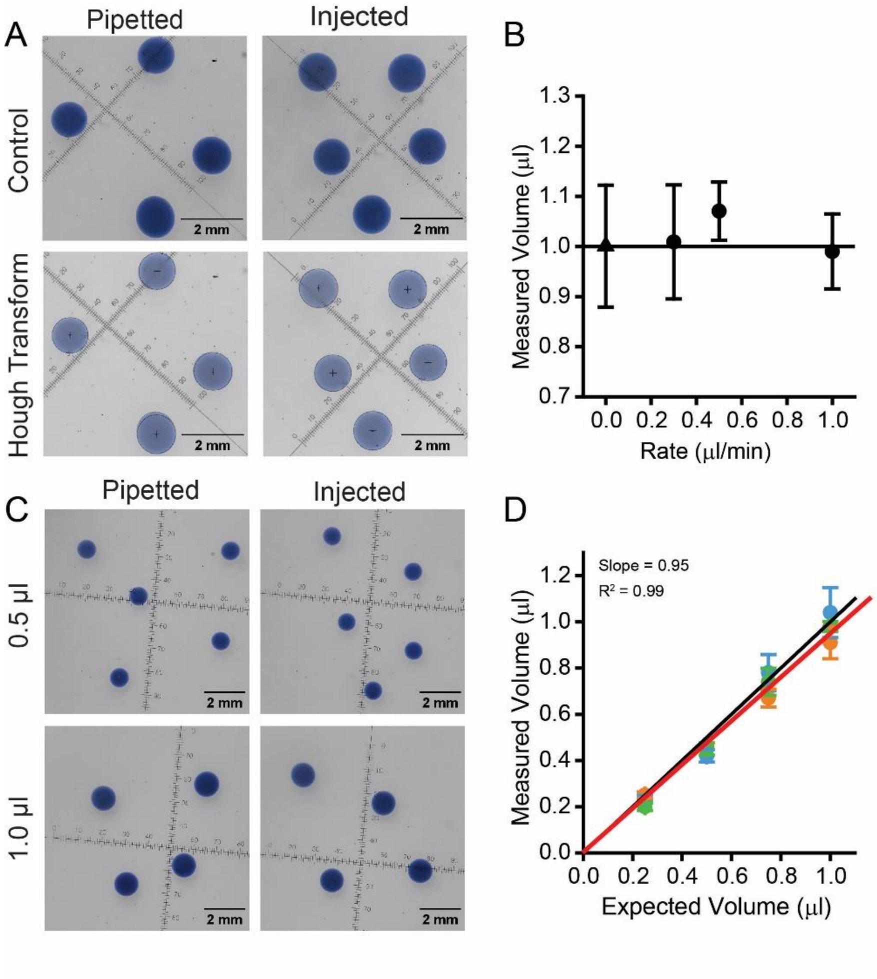

Sfigure 2: Calibration of pump and drug delivery system. (**A**) Raw images of micropipetted (left) and pump injected (right) trypan blue (0.4%) diluted 1:9 with distilled water 1ml volumes into silicone oil. Panels below are the Hough transform used to detect the edges of the droplets and the scale bar was photographed simultaneously with the droplets to serve as calibration for absolute magnification through the dissecting microscope. (**B**) plots the measured volume, calculated from images in panel **A** against the pump rate, with error bars being SD (n=5). The hand pipetted spheres are represented by the triangle presented at the rate = 0 point on the x-axis. The fitted line has a slope of 0. (**C**) presents pipetted and injected spheres at 2 sizes, 0.5 and 1.0 mls using the same solutions as in panel **A**. (**D**) plots the results from independent experiments. Each symbol is the average from 5 spheres with the error bar being SD. The black line is Y=X, the red line is fitted and has a slope of 0.95 and r^2 of 0.99.

Supplemental Figure 3

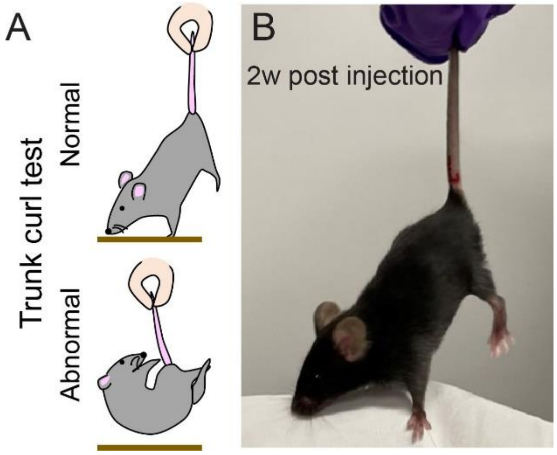

Sfigure 3. Trunk-curl test after artificial perilymph (AP) injection. (**A**) Illustration of the truck-curl test showing normal and abnormal vestibular responses. (**B**) Representative image of a mouse undergoing the truck-curl test at two weeks after artificial perilymph injection.

Supplemental Figure 4

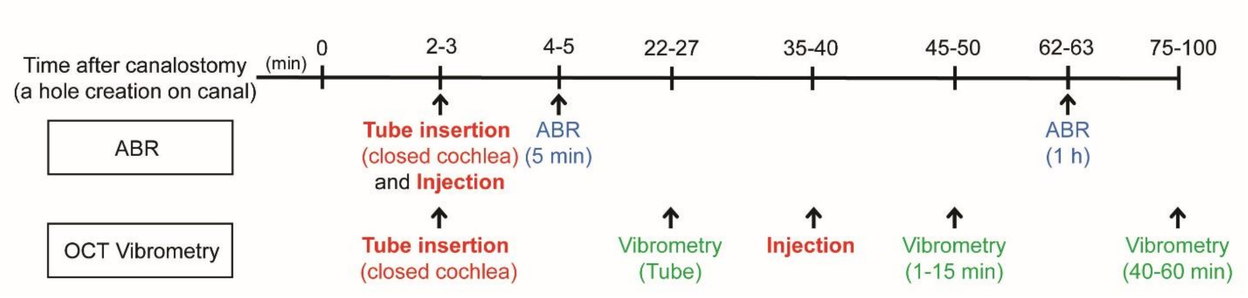

Sfigure 4. A diagram of the timeline for the experiments. Auditory brainstem responses (ABR) and vibrometry based on optical coherence tomohrapy (OCT) are performed at different time points.

Supplemental Movie Captions

Smovie 1. Vestibular behavioral assessment after artificial perilymph (AP) injection. Representative movie of a mouse (4x speed) shows no vestibular behavioral defect (head bobbing, head tossing, head tilting, and circling) at two weeks after artificial perilymph injection.

Smovie 2. Swimming test after artificial perilymph (AP) injection. Representative movie of a mouse (4x speed) shows no vestibular defect in swimming test. The scores of the swimming tests are zero (i.e., normal swimming) in all mice at two weeks after artificial perilymph injection based on criterion previously determined (39).

Smovie 3. In vivo real-time imaging of gold nanoparticles (GNPs) during PSCC injection. In vivo optical coherence tomography (OCT) recording of GNPs reveals that PSCC injection enters the perilymphatic space.
